# Progression of Gastric Acid Production in Preterm Neonates: Utilization of *In-vitro* Method

**DOI:** 10.3389/fped.2018.00211

**Published:** 2018-08-07

**Authors:** Murali R. Palla, Shashidhar Harohalli, Tim N. Crawford, Nirmala Desai

**Affiliations:** ^1^Pediatrics, University of Kentucky, Lexington, KY, United States; ^2^Division of Pediatric Gastroenterology, New Hampshire's Hospital for Children, Manchester, NH, United States; ^3^Department of Population and Public Health Sciences, Wright State University, Dayton, OH, United States

**Keywords:** preterm infants, gastric pH, progression of acidity, reflux, *Invitro* method

## Abstract

**Background:** Limited studies are done regarding ability to produce gastric acid in preterm infants and most studies used *in vivo* method of assessing gastric pH.

**Objectives:** To assess the feasibility of using an *in vitro* method of measuring gastric pH in babies ≤ 28 weeks gestational age (GA) and determine whether changes in gastric pH differ with gestational age, mode of delivery, and use of antenatal steroids.

**Design/Methods:** Prospective study that enrolled extremely low birth weight (ELBW) babies. Gastric aspirate collected before feeding. *In vitro* testing of gastric aspirates for pH were done on days of life 1, 3, 5, 7, 14, and 28 by using pH electrode. The pH was measured on each sample in triplicate, mean calculated and used for data analysis. Stastical methods included descriptive statistics, *t*-tests and repeated measures ANOVA.

**Results:** 29 subjects ≤ 28 weeks or birth weight ≤ 1,000 g were enrolled. No significant change was noted in pH measurements over time. Antenatal steroids and mode of delivery did not affect gastric acid pH.

**Conclusion:** The *in vitro* method for gastric pH measurements is non-invasive and affords more frequent testing. It would be useful in studying various conditions that may affect gastric pH.

## Introduction

The use of antacid agents to treat gastroesophageal reflux in preterm infants is common. The gold standard for pH measurement is relatively invasive and can only be performed in larger infants. Hence, the vast majority of neonatologists start acid suppression empirically without a diagnostic study or work up (1). However, acid suppression in this patient population is not benign with rising evidence linking use with significant morbidities such as sepsis and necrotizing enterocolitis (1). Acid suppression should be very selective and limited to symptomatic patients with increased gastric acidity.

The reference values and natural progression of gastric pH in extremely preterm infants is poorly studied. This is a prospective study in extremely preterm babies by using an *in vitro* method of assessing gastric pH changes during the first month of life. Earlier studies used *in vivo* method to study development of gastric secretory process in preterm infants in assessing gastric pH (2–4). At birth gastric pH is >2 and decreases as the stomach begins to secrete acid. Most studies indicate that acid secretion increases from birth to day 10, then declines again over the next 20 days. Report show that antenatal steroid use is associated with increased gastro-esophageal reflux in preterm neonates and pH is significantly lower after vaginal delivery than after cesarean section (5, 6). Earlier studies have used continuous pH measurements, but in a small number of patients. The objective of the study were to assess the feasibility of using an *in vitro* method of measuring gastric pH in babies ≤ 28 weeks ‘gestational age (GA) and determine whether changes in gastric pH differ with GA, mode of delivery, and use of antenatal steroids.

## Methods/design

This was a prospective study (between 2010-2012) that was approved by the Institutional Review Board and complied with applicable HIPPA standards. Informed consent was obtained from the parent(s) prior to enrollment. Inclusion criteria included admission to the neonatal intensive care unit, GA ≤ 28 weeks and birth weight (BW) ≤ 1,000 g, and infants had nasogastric (NG) tube as part of standard care. Exclusion criteria were major congenital anomalies (such as congenital heart disease, gastroschisis, and omphalocele), non-English speaking parents, or parents < 18 years of age. Gastric aspirate was collected through NG tube before feeding on days of life 1, 3, 5, 7, 14, 21, and 28. The pH measurement stopped at 28 days of life as some infants will already be on full feeds without NG tube. The specimen was collected by research staff, transported on ice, stored, and tested at a later time. *In vitro* testing of gastric aspirates for pH was done, using a solid state PHR-46 Micro combination pH electrode (Lazar Research Laboratories, Inc., Los Angeles, CA, USA); this instrument has been used in earlier studies to detect pH of body fluids (7). We utilized a neutral pH solution to calibrate the electrode before each measurement. The pH was measured on each sample in triplicates.

### Statistical analysis

The mean pH measured on each sample in triplicate was calculated and used for data analysis. Coefficient of variation (CV) was employed to determine percent variability among triplicate measurements. Data was analyzed using SAS v9.2 (Cary, NC). Means and standard deviations (SD) were calculated from continuous variables. To determine differences in gastric pH by GA, antenatal steroid use and mode of delivery for each time period, independent two sample *t*-tests were employed. Repeated measure ANOVA was employed to determine the effect GA on gastric pH over time.

## Results

Twenty-nine subjects (19 males and 10 females) were enrolled. All were ≤ 28 weeks and had BW ≤ 1,000 g. Of these, 12 were <26 weeks and 17 were ≥ 26 weeks GA. Eleven had BW of < 800 g and 18 were ≥ 800 g. The mean (SD) of birth weight and gestational age was 801 (196.8) grams and 26 5/7 (1.61) weeks respectively. Twenty-one babies received complete course of steroids, 6 partial and 2 no steroids. Mode of delivery was C-section in twenty-three and vaginal in six. The first feeding occurred at mean day of life 3.

Table 1 shows mean (SD) of serial gastric pH measurements with the *in vitro* method for each day over time by GA groups (<26 and ≥26 weeks). The mean Coefficient of variation (CV) for the measurements across the time period was 1.16%. Table 2 shows that antenatal steroid use and mode of delivery did not affect gastric pH.

**Table 1:** Mean pH and Standard deviation (SD) over period of time by Gestational age (GA).

**Characteristics**	**Day 1**	**Day 3**	**Day 5**	**Day 7**	**Day 14**	**Day 21**	**Day 28**
All infants	5.28 (2.66)	4.38 (1.17)	4.36 (1.64)	3.65 (1.48)	3.92 (1.39)	4.17 (1.16)	4.69 (1.22)
GA ≥ 26 weeks (*N* = 17)	5.25 (3.00)	4.5 (1.79)	4.34 (1.69)	3.29 (1.49)	3.9 (1.59)	4.1 (1.23)	4.56 (1.34)
GA < 26 weeks (*N* = 12)	5.33 (2.17)	4.21 (1.60)	4.39 (1.56)	4.17 (1.47)	3.94 (1.10)	4.27 (1.05)	4.87 (1.06)

**Table 2:** Mean pH and Standard deviation (SD) over period of time by antenatal steroid use and mode of delivery.

**Characteristics**	**Day 1**	**Day 3**	**Day 5**	**Day 7**	**Day 14**	**Day 21**	**Day 28**
No antenatal Steroids (N = 8)	5.03 (1.49)	4.74 (1.01)	4.08 (1.13)	3.44 (1.03)	4.28 (1.29)	4.60 (0.73)	5.17 (0.75)
Yes antenatal Steroids (N = 21)	4.39 (2.49)	4.32 (1.93)	4.54 (1.72)	3.70 (1.67)	3.80 (1.42)	4.03 (1.22)	4.50 (1.33)
C-section (N = 23)	4.92 (2.67)	4.48 (1.78)	4.37 (1.69)	3.70 (1.56)	4.11 (1.35)	4.13 (1.15)	4.59 (1.26)
Vaginal (N = 6)	4.97	4.36 (1.3)	3.95 (1.09)	3.30 (1.41)	3.32 (1.39)	4.33 (1.15)	5.04 (1.10)

## Discussion

In this study we were able to measure serially gastric pH in preterm babies by a non-invasive method. Most studies measured gastric pH by using a 24 h pH probe for shorter duration; i.e., a few days (2, 3, 8, 10). Unlike other studies, we were able to measure gastric pH not only on first day but intermittently over a longer period of time in critically ill preterm babies thus establishing the evolution of gastric acidity in ELBW infants.

Our study showed that gastric pH was acidic on day 1 and remained constant up to 4 weeks. An earlier study using *in vivo* method showed that there was a decrease in intragastric pH measured from day 1 to day 16 in infants with < 28 GA and showed that as the infants became more mature, both in terms of gestation and postnatal age, there was decrease in intragastric pH (2). In contrast, we found no difference in gastric pH between the GA groups over time.

Earlier study showed that babies who received antenatal steroids had clinical evidence of gastro-esophageal reflux and the gastric pH was acidic. They speculated that repeated use of antenatal steroids disrupt the normal neuronal maturation of the esophageal body and lower esophageal sphincter, thereby predisposing the infant to an increased incidence of gastro esophageal reflux (5). Studies on gastro esophageal reflux most often employed the esophageal pH measurement by a catheter or measurement of esophageal impedance by multiple intraluminal impedance (MII) catheter (9, 10). Our study showed no difference in gastric pH in relation to antenatal steroid use, though most of the babies in our study had complete course antenatal steroids and only 8 had partial or no steroids and our study was not designed to address the potential of *in vitro* pH measurement technique to be employed to study gastro esophageal reflux.

Miclat et al. reported that gastric pH was lower after vaginal delivery compared to cesarean section delivery. This was attributed to increased response to stress associated with labor and vaginal delivery as gastric secretion is regulated by both nervous and hormonal mechanisms, the nervous regulation being effected primarily through the parasympathetic fibers of the vagus nerves. They postulated that pressure phenomena, most likely on the fetal head, initiate vagal stimulation and gastric secretion (6). There was no difference in gastric pH between cesarean section and vaginal delivery in our study.

This study shows feasibility of *in vitro* gastric pH measurement, a simple non-invasive bedside test which allows serial measurements even in sick babies. Gastric pH values in preterm babies showed functional maturation and pH was acidic on day 1 and remained relatively constant throughout study period. These findings indicate that preterm infants can produce enough acid to lower gastric pH even from day 1 and there is not a progressive decline indicating that maturity appears to be attained at birth even in low birth weight preterm neonates < 28 weeks gestational age. We believe the method utilized in our study offers the advantage of non-invasive monitoring. In contrast, the current gold standard for pH measurement is relatively invasive and mostly utilized with advanced postnatal age. The proposed non-invasive method can be utilized in smaller extremely preterm infants and affords more frequent testing and serial measurement, spread over days and weeks. This method is valuable as it can guide clinician's decision making and limit acid suppression use in extremely preterm infants.

### Study limitations

We limited our enrollment to very premature ELBW infants; therefore we have no results on pH by the *in vitro* method in more mature infants. Another limitation is that we did not collect data on the causes of prematurity (such as chorioamnionitis and pre-eclampsia), type of feeds, apnea/bradycardia events and medications. Our number of infants studied was small; a larger number will be needed to apply this *in vitro* method in practice. Finally, we also did not perform comparison studies of this method and the gold standard *in vivo* method.

## Conclusion

The *in vitro* method of measuring gastric acid pH may be a useful noninvasive bedside tool in NICU. However further studies need to be done to establish its application in clinical practice.

## Ethics statement

The study was approved by the Institutional Review Board at the University of Kentucky.

## Author contributions

All authors listed have made a substantial, direct and intellectual contribution to the work, and approved it for publication.

### Conflict of interest statement

The authors declare that the research was conducted in the absence of any commercial or financial relationships that could be construed as a potential conflict of interest.
